# B Cell Antigen Receptor Signaling and Internalization Are Mutually Exclusive Events

**DOI:** 10.1371/journal.pbio.0040200

**Published:** 2006-05-30

**Authors:** Ping Hou, Elizabeth Araujo, Tong Zhao, Miao Zhang, Don Massenburg, Margaret Veselits, Colleen Doyle, Aaron R Dinner, Marcus R Clark

**Affiliations:** 1Department of Medicine and Section of Rheumatology, University of Chicago, Chicago, Illinois, United States of America; **2**Committee on Immunology, University of Chicago, Chicago, Illinois, United States of America; **3**Department of Chemistry, University of Chicago, Chicago, Illinois, United States of America; **4**Institute for Biophysical Dynamics, University of Chicago, Chicago, Illinois, United States of America; **5**James Franck Institute, University of Chicago, Chicago, Illinois, United States of America; Scripps Research InstituteUnited States of America

## Abstract

Engagement of the B cell antigen receptor initiates two concurrent processes, signaling and receptor internalization. While both are required for normal humoral immune responses, the relationship between these two processes is unknown. Herein, we demonstrate that following receptor ligation, a small subpopulation of B cell antigen receptors are inductively phosphorylated and selectively retained at the cell surface where they can serve as scaffolds for the assembly of signaling molecules. In contrast, the larger population of non-phosphorylated receptors is rapidly endocytosed. Each receptor can undergo only one of two mutually exclusive fates because the tyrosine-based motifs that mediate signaling when phosphorylated mediate internalization when not phosphorylated. Mathematical modeling indicates that the observed competition between receptor phosphorylation and internalization enhances signaling responses to low avidity ligands.

## Abstract 2

The B cell receptor (BCR) is responsible for both the internalization of pathogens by B cells and for B cell activation signaling; internalization and activation signaling are mutually exclusive events for an individual BCR.

## Introduction

The recognition of polyvalent antigens by the B cell antigen receptor (BCR) initiates a complex web of signaling events that determine cellular responses [ 1]. Polyvalent antigen also induces the rapid internalization of engaged receptors which is required for the effective presentation of antigen-derived MHC class II restricted peptides to T cells [ 2]. As important as these two processes are, the relationships between them are poorly understood.

Through the work of numerous investigators, a clear picture of initial signaling through the BCR has emerged [ 3]. Receptor engagement induces the phosphorylation of tyrosines contained within conserved motifs (immunoreceptor tyrosine-based activation motifs or ITAMs) in the cytosolic tails of the receptor constituents Igα and Igβ [ 4, 5]. The initial phosphorylation of the ITAM tyrosines is mediated by both Syk [ 6, 7] and members of the Src-family of tyrosine kinases (SFTKs) including Lyn, Fyn, and Blk [ 6, 8]. Once phosphorylated, Igα/Igβ ITAMs serve to recruit and activate the tyrosine kinase Syk [ 9– 11]. The SFTKs can also be activated by recruitment to the receptor [ 12], although dephosphorylation by CD45 is likely to be the major mechanism of SFTK activation [ 13, 14]. Once activated, the SFTKs and Syk initiate distinct and inter-related signaling pathways. SFTKs are required for the activation of NFκB [ 7] and serve to phosphorylate additional important signaling substrates such as CD22 [ 15] and BAM32 [ 16– 18]. Syk phosphorylates BLNK (also termed BASH or SLP65) [ 19– 22], a scaffolding molecule that is recruited to the BCR through a unique phosphorylated non-ITAM tyrosine in the Igα cytosolic tail [ 23, 24]. BLNK coordinates the assembly and activation of a receptor-retained signalsome containing PLCγ2, Vav, Btk, Nck, and Grb2 [ 25].

Concurrent with signal initiation, the majority of aggregated BCR complexes are rapidly cleared from the cell surface. The endocytosis of receptor-bound antigen is the first in a series of signaling-mediated events that ensures that low affinity antigens are efficiently captured, processed, and presented to cognate T cells [ 26]. These include the rapid sorting of internalized antigens to late endosomal antigen processing compartments [ 27, 28] and the acidification and remodeling of these targeted late endosomes [ 29, 30]. BCR signaling also enhances the synthesis of MHC class II [ 31] and the co-stimulatory molecules B7–1 and B7–2 [ 32]. In the absence of BCR-mediated activation, resting B cells cannot productively capture and present antigen to T cells.

While the necessity of BCR internalization for antigen presentation is clear, its relationship to signal propagation is largely unknown. Recent observations in clathrin-deficient DT40 cells [ 33] suggest that internalization may extinguish receptor signaling. However, studies of BCR internalization using pharmacological inhibitors have yielded seemingly contradictory results [ 18, 34]. In contrast, internalization of the growth factor receptors, such as epidermal growth factor receptor, may be required for the efficient activation of selected signaling molecules including Erk [ 35, 36].

Much of the uncertainty regarding the impact of BCR endocytosis on signaling is due to our incomplete knowledge of the mechanisms governing internalization. BCR endocytosis requires clathrin and actin polymerization [ 33, 37– 39]. It also requires activation of a SFTK [ 18, 40], which may function to phosphorylate clathrin heavy chain [ 39]. BAM32, which is also phosphorylated by the SFTKs, is required for efficient receptor internalization [ 18]. This scaffolding molecule probably contributes to endocytosis by regulating Rac1 and the remodeling of actin. The molecular linkage between these signaling pathways, clathrin and the BCR remains enigmatic. It has been postulated that lipid rafts are the intermediate between the BCR and clathrin [ 39]. However, BCR complexes can be efficiently internalized without segregating to lipid rafts [ 41]. Within the BCR complex, the multiple tyrosine-based motifs could serve as binding sites for clathrin adaptors [ 42]. However, there has not been a systematic analysis of their contribution to receptor endocytosis [ 43, 44].

Herein, we demonstrate that non-ITAM tyrosines, and to a lesser degree the ITAM tyrosines, determine BCR internalization. This finding suggested that phosphorylated, and therefore actively signaling receptor complexes, are preferentially retained on the cell surface. This prediction was confirmed in both biochemical and confocal microscopy studies. Based on these results, we developed a mathematical model of the observed exclusive relationship between receptor internalization and phosphorylation that was then compared to a hypothetical non-exclusive model. Analysis of the exclusive model indicated that it could account for previous seemingly contradictory observations on the relationship between BCR internalization and signaling. Furthermore, it revealed that retaining phosphorylated BCRs on the cell surface should preferentially enhance signaling responses to low avidity ligands.

## Results

### The Tyrosines within Igα Are Required for Internalization

Tyrosine-based motifs consisting of YXXφ are well described binding sites for AP (adaptor protein)-2, and other adaptors, which mediate receptor endocytosis [ 45]. Surprisingly, there has been no systematic examination of the role of the Igα/Igβ cytosolic tyrosines in BCR internalization. Therefore, we undertook such an analysis with a focus on Igα because it is the main signaling chain of the BCR [ 46, 47], and it has been implicated in constitutive receptor internalization [ 44].

**Figure 1:**
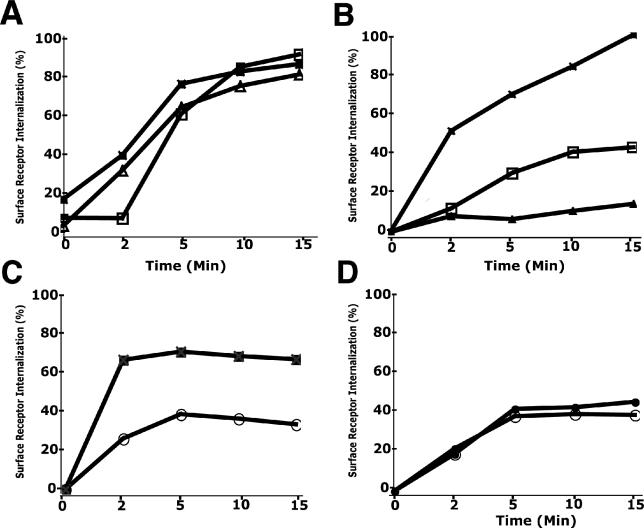
BCR Endocytosis Is Determined by Cytosolic Tyrosine-Based Motifs A20IIA1.6 cells expressing chimeras containing the cytosolic tail of either wild-type Igα or Igα in which the ITAM or non-ITAM tyrosines were mutated were assayed for endocytosis. (A) Comparison of Igα ^wt^ (solid squares) to Igα in which ITAM tyrosines were mutated to phenylalanines (Igα ^YΔF182,193^) (open triangles) or alanines (Igα ^YΔA182,193^) (open squares). (B) Comparison of the internalization of Igα ^wt^ (solid squares), a cytosolic tail truncation of Igα (ΔT, open triangles), and a ITAM/non-ITAM Igα mutant (Igα ^YΔA176,182,193,204^, open squares). (C and D) Igα ^wt^ (C) or Igα ^YΔA176,182,193,204^ (D) were assayed for endocytosis in the presence (closed symbols) or absence of Latrunculin A (open symbols).

To simplify our initial analysis, we chose to examine the cytosolic tail of Igα in isolation as a chimera with the extracellular and transmembrane domains of human platelet derived growth factor receptor (PDGFR) [ 47, 48]. For these experiments, we derived chimeras containing several Igα cytosolic mutants. DNA fragments encoding each PDGFRβ/Igα chimera were cloned into a retroviral vector (MIGR1) containing an IRES/EGFP cassette. The murine FcR ^−^ B cell lymphoma A20IIA1.6 was infected with viral stocks encoding each chimera, and cell populations expressing similar surface levels of each receptor were isolated by flow cytometry (unpublished data). We first compared the internalization of wild-type Igα (Igα ^wt^) to Igα in which the ITAM tyrosines were mutated to either phenylalanines (Igα ^YΔF182,193^) or alanines (Igα ^YΔA182,193^). Conservative mutations to phenylalanines prevent Syk recruitment [ 9] but should not interfere with the recruitment of putative adaptors involved in receptor internalization [ 49]. In contrast, mutations to alanine should prevent the recruitment of both Syk and any adaptor molecules [ 45, 50]. Endocytosis of each ligated chimera was assayed by flow cytometry and the percent of internalized receptor was plotted as a function of time. As can be seen in Figure 1A, mutation of the ITAM tyrosines to phenylalanines had little effect on receptor endocytosis while mutation to alanines slightly delayed receptor internalization ( *n* = 4). Endocytosis of the endogenous BCR on each test cell population was similar (unpublished data) confirming that there were no clonal differences in internalization capacity. These data indicate that the Igα ITAM tyrosines make a modest contribution to receptor internalization.

We next derived and analyzed additional Igα mutants to examine if the non-ITAM tyrosines contributed to receptor internalization (Igα ^YΔA176,182,193,204^) and if the cytosolic tail contained other functional domains (Igα ^ΔC^). As can be seen in Figure 1B, additive mutation of the non-ITAM tyrosines inhibited both the rate and magnitude of internalization by approximately 70%. Single additive mutation of Y ^176^ (Igα ^YΔA176,182,193^) or Y ^204^ (Igα ^YΔA182,193,204^) revealed that each contributed to efficient receptor internalization (unpublished data). As mutation of the non-ITAM tyrosines alone has little effect on receptor internalization [ 24], we conclude that both the ITAM and non-ITAM tyrosines contribute to BCR internalization.

Interestingly, truncation of the Igα cytosolic tail completely inhibited internalization. These data indicate that additional, unidentified domains within the cytosolic tail contribute to BCR internalization. However, as demonstrated below, it is the tyrosine-based motifs that determine which BCR complexes are internalized.

Recent observations suggest that actin cytoskeleton remodeling is important for BCR internalization [ 18, 51]. Therefore, we assayed whether tyrosine-mediated Igα internalization was dependent or independent of actin polymerization. Cells expressing Igα ^wt^ ( Figure 1C) or Igα ^YΔA176,182,193,204^ ( Figure 1D) were stimulated as above in the presence of Latrunculin A. As can be seen, Latrunculin A inhibited the internalization of Igα ^wt^ but had no effect on the internalization of Igα ^YΔA176,182,193,204^. These data indicate that tyrosine-mediated internalization depends on actin polymerization. The mechanisms underlying the residual tyrosine-independent and actin-independent receptor internalization are not known.

### Phosphorylated BCR Complexes Are Retained on the Cell Surface

**Figure 2:**
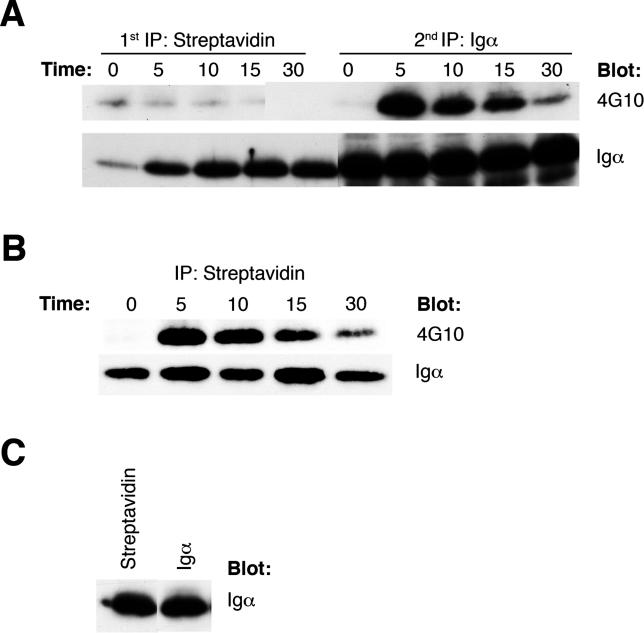
Following BCR Stimulation, Phosphorylated Igα Is Retained on the Cell Surface (A) A20IIA1.6 cells were surface-biotinylated with Sulfo-NHS-SS-Biotin on ice, stimulated with anti-IgG for the indicated times (min) and then biotin was stripped from the cell surface. Lysed samples were precipitated sequentially with streptavidin and then anti-Igα antibodies. Parallel samples were then immunoblotted with anti-phosphotyrosine (4G10) or anti-Igα antibodies. (B) Cells were labeled and stimulated as above except that biotin was not stripped from the cell surface. Streptavidin precipitations were then sequentially immunoblotted with 4G10 and anti-Igα antibodies. (C) Resting surface-biotinylated cells were directly lysed and sequentially precipitated with streptavidin and then anti-Igα antibodies. Precipitations were Western blotted with anti-Igα antibodies.

With other receptors that depend upon tyrosine-based motifs for internalization, phosphorylation at these residues impedes endocytosis [ 49, 52]. Therefore, our data predicted that following engagement, phosphorylated BCR complexes would be preferentially retained on the cell surface while non-phosphorylated complexes would be internalized. To test this prediction, resting wild-type A20IIA1.6 cells were surface labeled on ice with Sulfo-NHS-SS-Biotin, a reagent that attaches biotin to surface proteins through a linker containing a thiol-cleavable bond. Cell aliquots were either left unstimulated or stimulated through the BCR for the indicated times at 37 ^°^C. Cells were then treated on ice with reduced glutathione which is a cell impermeable reducing agent that strips biotin from surface, but not internalized, receptor complexes. Lysates from each sample were sequentially precipitated with strepavidin-Sepharose followed by anti-Igα antibodies. Precipitations were resolved by SDS-PAGE, transferred to membranes and then probed with either anti-phosphotyrosine (4G10) or anti-Igα antibodies ( Figure 2A). In the strepavidin precipitations, little Igα was precipitated from resting cells, but progressively more material was detected over time, reflecting progressive internalization of the stimulated BCR. Although Igα was readily detected in these precipitations, there was essentially no phosphorylated Igα detected. In contrast, phosphorylated Igα was readily detected in subsequent anti-Igα immunoprecipitations ( *n* = 5).

The results in Figure 2A could have been obtained if surface-labeled complexes were not phosphorylated following BCR ligation or if they were not efficiently precipitated with strepavidin. Therefore, cells were stimulated as in Figure 2A but without subsequent incubation with glutathione. Stepavidin precipitations were then sequentially Western blotted with 4G10 followed by anti-Igα antibodies. As can be seen in Figure 2B, phosphorylated Igα was readily detected in the strepavidin precipitations. To assess the efficiency of surface biotinylation, resting cells were surface-biotinylated, lysed, and then sequentially precipitated with strepavidin and anti-Igα antibodies ( Figure 2C). Approximately 50% of the detectable Igα was present in the strepavidin precipitations indicating that Igα was efficiently labeled on the cell surface.

### Direct Visualization of Phosphorylated BCR Complexes

**Figure 3:**
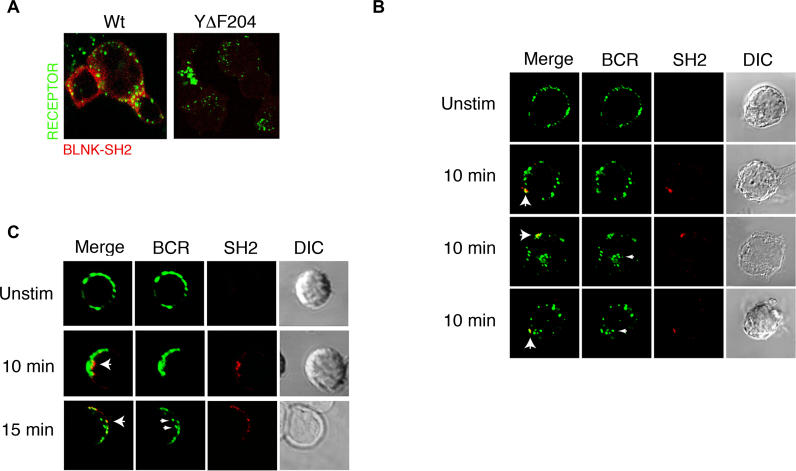
Direct Visualization of BCR Complexes Containing Igα Phospho-Y ^204^ (A) A20IIA1.6 cells expressing PDGFR chimeric receptors containing the cytosolic tail of either Igα or Igα ^YΔF204^ were stimulated through the chimera with FITC-conjugated antibodies for 10 min, fixed, permeabilized, and counterstained with PE-BLNK-SH2 (SH2). Cells were then visualized using confocal microscopy. (B) BLNK ^kd^ cells were stimulated with Cy5-conjugated anti-IgG antibodies for 10 min. Samples were then fixed, permeabilized, counterstained with PE-BLNK-SH2, and visualized by confocal microscopy. Typical results are shown ( *n* = 150 cells from three independent experiments). (C) Purified BLNK ^−/−^ splenic B cells were stimulated with FITC-conjugated anti-IgM F(ab) _2_ fragments for 10 or 15 min and then fixed, permeabilized, counterstained with PE-BLNK-SH2, and visualized by confocal microscopy. Small arrowheads indicate internalized non-phosphorylated BCR complexes while large arrowheads indicate locations of phosphorylated surface BCRs.

Previously, we had demonstrated in pull-down and far Western assays that the BLNK SH2 domain bound directly and specifically to phosphorylated Y ^204^ Igα [ 23]. Given the specificity of this interaction, we reasoned that a GST fusion protein containing the BLNK-SH2 domain could be used to visualize BCR receptor complexes in which Igα Y ^204^ was phosphorylated. To test this idea, we first coupled a GST fusion protein containing the BLNK-SH2 domain [ 23] to phycoerythrin (PE). A20IIA1.6 cells expressing PDGFR chimeric receptors containing the cytosolic tail of either Igα or Igα ^YΔF204^ were stimulated through the chimera with FITC-conjugated antibodies for 10 min; fixed, permeablized, and counterstained with PE-BLNK-SH2. Staining was only observed in those cells stimulated with a receptor containing an intact Igα cytosolic tail ( Figure 3A). Although specific staining was observed, there was also significant background. We postulated that this could be due to competition with endogenous BLNK. Therefore, we derived A20IIA1.6 cells in which BLNK had been knocked down with shRNA (BLNK ^kd^, Figure S1).

BLNK ^kd^ cells were stimulated with Cy5-conjugated anti-IgG antibodies for 10 min. Samples were then fixed, permeabilized, and counterstained with PE-BLNK-SH2. As can be seen, there was no PE-BLNK-SH2 staining in unstimulated cells ( Figure 3B). In contrast, specific co-localization with surface BCR complexes was seen in stimulated cells. Only surface-retained BCR patches were phosphorylated while no internalized BCR complexes were detectably phosphorylated ( *n* = 150 cells, with internalized BCR complexes from three independent experiments). Similar results were obtained when cells were stimulated for 15 min while by 30 min after stimulation, phosphorylated complexes were no longer detectable (unpublished data).

We next sought to extend our studies to splenic B cells. In BLNK ^−/−^ mice, there is a partial block in B cell development [ 53]. However, peripheral IgM ^+^ B cell populations accumulate after birth [ 53, 54] (unpublished data). Therefore, purified BLNK ^−/−^ splenic B cells were stimulated with FITC-conjugated anti-IgM F(ab) _2_ fragments for 10 or 15 min and then fixed, permeabilized, and counterstained with PE-BLNK-SH2. In contrast to A20IIA1.6 cells, ligation of the BCR on BLNK ^−/−^ splenic B cells rapidly induces a single cap containing most of the ligated BCR complexes ( Figure 3C). However, only a fraction of the receptors in each cap were phosphorylated and no internalized BCR complexes were demonstrably phosphorylated as measured by BLNK-SH2 binding ( *n* = 100, four experiments). From these studies, we conclude that following BCR stimulation, phosphorylated BCRs are retained on the cell surface and are not internalized.

**Figure 4:**
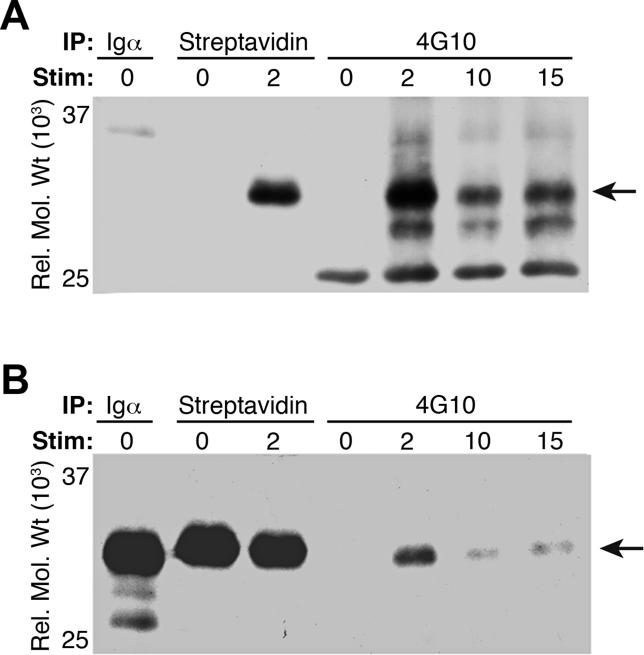
Only a Small Fraction of Igα Is Phosphorylated following BCR Stimulation Aliquots of wild-type A20IIA1.6 cells were surface-biotinylated and then stimulated through the BCR for the indicated times. Following lysis, cells were precipitated with strepavidin, 4G10, or anti-Igα antibodies. Precipitations were resolved by SDS-PAGE, transferred to nylon membrane and immunoblotted sequentially with 4G10 (A) and then anti-Igα antibodies (B). Results of a typical experiment are presented ( *n* = 3). Arrows denote Igα.

Interestingly, only a minority of surface BCRs were phosphorylated. This is most clearly seen in the A20IIA1.6 cell line in which only 10%–20% of surface patches, or approximately 1 to 2 surface patches per cell, contained detectably phosphorylated BCR complexes. These results suggest that only rare surface BCR complexes initiate signaling and that these then segregate into common patches. Therefore, we next quantitated the fraction of surface Igα that was phosphorylated following BCR engagement. Aliquots of wild-type A20IIA1.6 cells were surface-biotinylated and then stimulated through the BCR for the indicated times ( Figure 4). Following lysis, cells were precipitated with anti-Igα antibodies, strepavidin, or 4G10. Precipitations were resolved by SDS-PAGE, transferred to membranes, and immunoblotted sequentially with 4G10 ( Figure 4A) and then anti-Igα antibodies ( Figure 4B). Precipitation with either strepavidin or anti-phosphotyrosine antibodies readily captured phosphorylated Igα ( Figure 4, upper panel) with the 4G10 immunoprecipitations being slightly more efficient. As expected, phosphorylation of Igα was greatest at 2 min with substantial decay at 10 and 15 min. To determine what fraction of surface Igα was phosphorylated, membranes were stripped and immunoblotted with anti-Igα antibodies. As can be seen, precipitation with either anti-Igα antibodies or strepavidin precipitated readily detectable amounts of Igα. In marked contrast, very little Igα was present in the corresponding 4G10 immunoprecipitation. Comparison of the Igα 32-kDa band densities at the 2-min stimulation time point revealed that 5% −10% of surface Igα was phosphorylated following BCR stimulation (typical results, *n* = 4). As the 4G10 immunoprecipitations were more efficient than the strepavidin precipitations, this may be a slight overestimation. Regardless, these results are consistent with those in Figure 3B indicating that signaling is propagated by a small minority of surface-retained BCR complexes.

### Inhibiting BCR Endocytosis Has a Modest Effect on Signal Propagation

Given that phosphorylated, and therefore signaling, receptor complexes are normally retained on cell surface, we predicted that inhibiting receptor internalization would not directly modulate receptor initiated signaling. Therefore, we expressed in A20IIA1.6 cells a dominant negative variant of dynamin (K44A), which prevents the scission of clathrin-coated vesicles [ 55] and has been used by others to examine the inter-relationships between internalization and signaling [ 51, 56]. As expected, retroviral-mediated expression of dn-dynamin-inhibited ligand induced BCR internalization by more than 90% [ 57]. These cells were used to assess the effect of internalization on BCR-initiated signaling.

**Figure 5:**
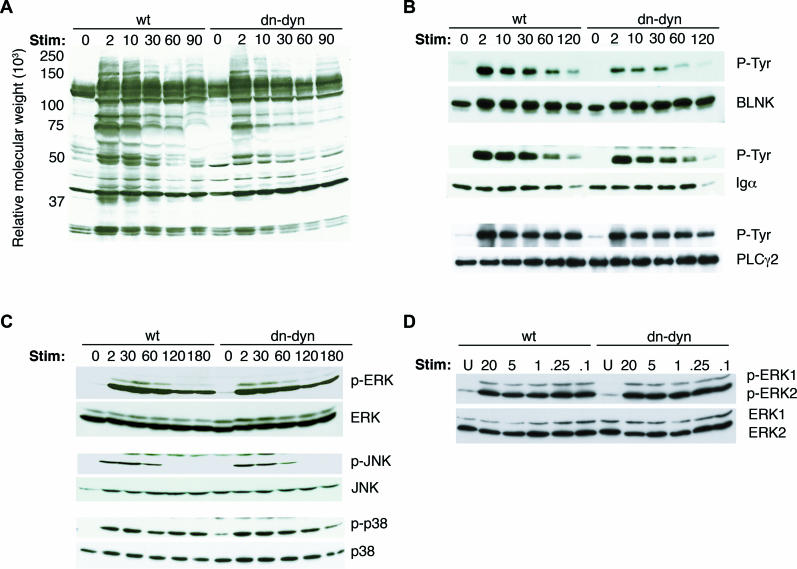
Inhibiting BCR Internalization Has Minimal Effect on Signaling In (A–C), A20IIA1.6 wild-type- or dn-dynamin-expressing cells were stimulated with anti-IgG for the indicated times (min). (A) Total cell lysates were immunoblotted with 4G10. (B) BLNK, Igα, or PLCγ2 immunoprecipitates were resolved by SDS-PAGE and then immunoblotted as indicated. (C) Total cell lysates were immunoblotted with phospho-specific antibodies to Erk, Jnk, or p38. Membranes were then stripped and reprobed with antibodies against Erk, Jnk, or p38 as indicated. (D) Cells were stimulated with decreasing amounts of anti-IgG (shown in μg/ml) for 3 min. Cells were then assayed for Erk activation as in (C).

Aliquots of cells expressing either dn-dynamin or empty vector were stimulated with anti-IgG antibodies for the indicated times, and total inductive tyrosine phosphorylation was assayed in Western blots with 4G10. As demonstrated in Figure 5A, blocking receptor internalization led to a modest decrease in global tyrosine phosphorylation without an appreciable change in activation kinetics. We then examined the inductive phosphorylation of specific substrates. Cell aliquots were stimulated as before and immunoprecipitates of Igα, BLNK, or PLCγ2 were resolved by SDS-PAGE and immunoblotted with 4G10. As can be seen in Figure 5B, there was an approximate 60% decrease in inductive BLNK phosphorylation at all time points examined. There was a more modest but reproducible ( *n* = 4) decrease in Igα phosphorylation in cells expressing dn-dynamin. In contrast, there was no significant change in PLCγ2 phosphorylation.

To assess if downstream signaling pathways were affected, cell aliquots were stimulated for the indicated times and total cell lysates were immunoblotted with either anti-phospho Erk, Jnk, or p38 antibodies. Blots were then stripped and immunoblotted with antibodies to detect the total amount of kinase in each lane. As can be seen in Figure 5C, there were no significant differences in the magnitude or duration of Erk, Jnk, or p38 activation. It is possible that subtle differences in mitogen-activated protein (MAP) kinase activation were obscured because the BCR was maximally stimulated in these experiments. However, when we titered the stimulating antibody over a 200-fold range, no differences were observed in Erk activation between dn-dynamin and control cells ( Figure 5D). Consistent with our model in which internalization per se does not directly attenuate BCR signaling, expression of dn-dynamin modestly inhibited some aspects of proximal BCR signaling and had little effect on distal MAP kinase activation.

### Modulation of BCR Signaling through the Internalization of Non-Phosphorylated Receptor Complexes

**Figure 6:**
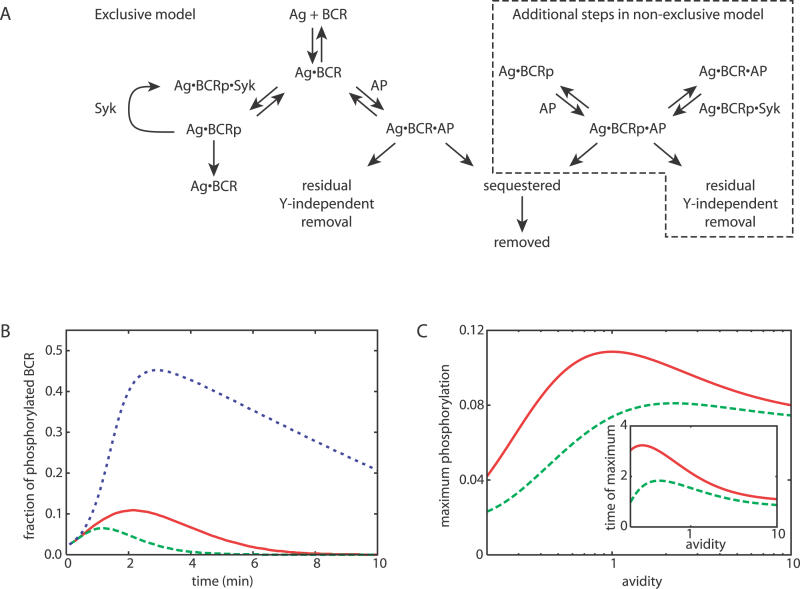
Mathematical Analysis of Interrelationships between Antigen Receptor Signaling and Internalization (A) Schematic representation of the exclusive and independent models of BCR phosphorylation and internalization. The independent model includes all the reactions in the exclusive model plus the additional ones indicated. Eliminating the sequestration reaction corresponds to clathrin deletion while eliminating the removal reaction corresponds to dn-dynamin (see discussion). (B) Extent of phosphorylation for wild-type (red), dn-dynamin (green), and clathrin mutant scenarios (blue). (C) Maximal signaling through the BCR, as assayed by Igα phosphorylation, as a function of ligand avidity. A comparison of exclusive (red) and independent (green) signaling and internalization models is provided (inset). Time to maximum BCR signal intensity as a function of ligand avidity as predicted for the exclusive (red) and independent (green) models. The parameters used to generate the figure are *k*_off_ = 2.0 min ^−1^, *k*_+a_ = *k*_–a_ = 4.0 min ^−1^, *k*_+s_ = 8.0 min ^−1^, *k*_–s_ = 4.0 min ^−1^, *k*_p_ = 10.0 min ^−1^, *k*_r_ = 5.0 min ^−1^, *k*_c_ = 4.0 min ^−1^, *k*_d_ = 2.0 min ^−1^, and *k*_g_ = 2.0 min ^−1^; *K*_i_ = *K*_M_ = 0.1 in units such that the initial BCR concentration is unity. The rate parameters were selected to give for the wild-type a maximum phosphorylation of about 10% at 2–3 min and decay on the order of 10 min. In the case of the non-exclusive model, we substitute either *k*_p_ = 12.0 min ^−1^ (faster phosphorylation), *k*_c_ = 2.0 min ^−1^ (slower sequester) or *k*_d_ = 8.0 min ^−1^ with *k*_g_ = 4.0 min ^−1^ (faster removal) to maintain the same maximum integrated phosphorylation to facilitate comparison. Data for scaled *k*_d_ and *k*_g_ are shown; scaled *k*_p_ and *k*_c_ give qualitatively similar behavior.

To explore the functional consequences of the observed competition between phosphorylation and internalization, we constructed a mathematical model ( Figure 6A). In this model, when the BCR engages antigen, one of two things can happen. One possible fate is that the engaged receptor is phosphorylated. For simplicity, the kinase-mediating receptor phosphorylation is Syk bound to another phosphorylated BCR (BCRp). The Src kinases also contribute to BCR phosphorylation [ 3]. However, recent observations suggest that this contribution is minor [ 6] (Clark, et al., unpublished data). Generating more phosphorylated BCRs, which can then bind and activate more Syk [ 9– 11], amplifies signaling through a positive feedback mechanism [ 6]. The other possible fate is that the engaged receptor associates with an AP which sequesters and then removes the receptor from the cell surface.

Sequestered BCRs are assumed to hinder the propagation of phosphorylation and are thus modeled as competitive inhibitors of the kinase, although they are not in fact expected to interact with it directly. Including this ansatz enables the model to account for some seemingly contradictory results that have been obtained using different strategies to inhibit BCR endocytosis. For example, in our experiments, expression of dn-dynamin slightly attenuated proximal signaling while deletion of clathrin has been reported to augment signaling [ 33]. Because dn-dynamin inhibits receptor internalization after receptors fated for endocytosis are already segregated into clathrin-coated pits [ 58], in the model the dn-dynamin mutant corresponds to inhibiting the removal of sequestered BCRs. As a result, receptors accumulate in the sequestered state, which limits the spread of phosphorylation. In contrast, in the absence of clathrin, coated pits cannot form, and the signal propagates freely through the receptor population ( Figure 6B).

We next examined how the observed mechanism of internalization might influence signaling responses to ligands of different avidity. For these studies, we compared the exclusive model to a conventional non-exclusive model in which phosphorylation and internalization were independent events (boxed reactions in Figure 6A; see Materials and Methods for a discussion of necessary modifications to the equations and parameters). As can be seen in Figure 6C, when signaling and internalization are mutually exclusive, signal intensities in response to low avidity ligands are preferentially enhanced as compared to the non-exclusive model. This effect derives from the fact that, in the former model, BCRp complexes are not removed from the surface, which makes it easier to overcome the action of phosphatases when relatively few receptors are engaged. On the other hand, at high avidities, the signal is lower relative to that predicted by the non-exclusive model. When phosphorylation and internalization are exclusive, the signal can only propagate to the relatively few BCRs not bound by an AP at any given time. The latter trend is more pronounced if one examines phosphorylation integrated over time (unpublished data). The essential difference between the low and high avidity regimes is that in the former, BCRp·Syk (the active catalyst) is limiting, while in the latter, Ag·BCR (the substrate) is limiting. These calculations thus indicate that the exclusive mechanism enhances the responses to low avidity ligands, which is likely to be important for initial detection in physiological contexts.

Interestingly, the exclusive model predicts that the time required to reach peak signal intensity will significantly vary with ligand avidities ( Figure 6C, inset). The effect is predicted to be especially pronounced at low avidities where the time to peak signal intensity should be significantly delayed. The physical basis for the delay is that selective endocytosis leaves BCRp on the surface to amplify the signal, but the signal spreads slowly due to the fact that relatively few BCRs are productively engaged by antigen. The non-exclusive model does not as readily support this scenario because BCRp does not persist on the surface. This predicted delay in peak-signaling intensity in response to low avidity ligands is a well-described feature of signaling through the BCR [ 59, 60].

## Discussion

In this paper, we provide direct evidence that once a BCR engages antigen it is either phosphorylated (and retained at the cell surface) or it is internalized. This was demonstrated using both biochemical and confocal microcopic techniques. The latter experiments made use of the unique binding specificity of the BLNK SH2 domain which allowed us to directly visualize the phosphorylated BCR. Within the sensitivity limits of our experiments, we never detected internalized phosphorylated receptors. Signaling and internalization are mutually exclusive events because the tyrosine-based motifs that mediate signaling when phosphorylated, mediate internalization when they are not phosphorylated.

It is well known that receptor endocytosis mediated by tyrosine-based motifs is subject to regulation by phosphorylation. The best-characterized example in lymphocytes is CTLA-4, where tyrosine phosphorylation of the cytosolic tail leads to the accumulation of CTLA-4 on the cell surface [ 49]. Surface expression of the adhesion molecule L1 is regulated in a similar manner [ 52].

However, in contrast to CTLA-4 and L1 in which single tyrosines determine receptor endocytosis, multiple tyrosines within Igα, and probably Igβ, contribute to internalization of the ligated receptor. Conservative mutations to phenylalanines had no detrimental effect on internalization while non-conservative mutations to alanine abrogated receptor internalization. These findings are consistent with recognition by an AP-2-like adaptor complex [ 42]. Both of the Igα ITAM tyrosines and the non-ITAM Y ^204^ tyrosine are within predicted AP-2 binding sites (YXXΦ, where Φ is a bulky hydrophobic amino acid)[ 61]. The second non-ITAM tyrosine at Y ^176^ has a glutamate at the Y + 3 position that should preclude recognition by AP-2 [ 62]. Furthermore, Y ^176^ does not appear to be phosphorylated in vivo following BCR ligation [ 23]. These latter observations suggest that the regulation of receptor internalization is complex and is not simply determined by the presence or absence of potential AP-2 binding motifs.

Indeed, even with mutation of all the Igα tyrosines there was some residual receptor internalization. It might have been predicted that this secondary mechanism of receptor internalization would lead to the endocytosis of some phosphorylated receptor complexes. However, in all of our biochemical ( Figure 2) and confocal ( Figure 3) studies, this was never observed. This might be due, in part, to the slower kinetics of these secondary endocytosis mechanisms. Another possibility is that our mutational analysis underestimated the importance of the tyrosines as determiners of internalization. In the activated receptor complexes, these tyrosines would not only be phosphorylated but would be bound to multimeric signaling complexes, which could limit the effectiveness of other secondary internalization mechanisms.

Previous studies examining the role of the Igα and Igβ ITAM tyrosines in receptor endocytosis have concluded that they did not make a significant contribution [ 43, 44]. Indeed, in our own studies, isolated mutation of the ITAM tyrosines had only a modest effect on receptor internalization. However, when mutated in combination with the non-ITAM tyrosines, endocytosis was significantly inhibited. The importance of the non-ITAM tyrosines had been overlooked until it was demonstrated that Y ^204^ was robustly phosphorylated in vivo and that it served to directly recruit the SH2 domain of BLNK [ 23]. In addition to a role in activating BLNK-dependent pathways, Y ^204^ is required for normal receptor endocytic trafficking [ 24] and for peripheral B cell selection (A.B. Cooper, L.D. Wang, and M.R. Clark, unpublished data). Therefore, the tyrosines within the BCR complex that mediate receptor internalization are of demonstrated functional and biological importance.

It has been suggested that lipid rafts may play a role in BCR endocytosis [ 39]. However, in immature B cells the activated BCR is excluded from lipid rafts, yet is efficiently internalized [ 41], indicating that lipid rafts are not obligatory. Cholesterol-rich microenvironments could play a role in modulating receptor endocytosis possibly by facilitating access to SFTKs [ 63]. Residency of the receptor in lipid rafts could also protect the receptor from dephosphorylation and subsequent endocytosis [ 64].

While conservative mutation of the ITAM tyrosines prevents the inductive tyrosine phosphorylation of cytosolic substrates [ 23], receptor internalization is unaffected ( Figure 2). The dispensability of the ITAM tyrosines for endocytosis is consistent with previous studies demonstrating that Syk activation is not required for internalization [ 9, 40]. Activation of a SFTK is necessary [ 18, 40], and evidence suggests that this can happen in the absence of an intact ITAM. It has been demonstrated that in vitro non-phosphorylated Igα can assemble with the SFTKs [ 12]. SFTKs can also be co-immunoprecipitated with the resting BCR in the absence of significant receptor phosphorylation [ 65]. These, and other observations [ 6, 7], indicate that the BCR may active the SFTKs and Syk through different mechanisms, and that each kinase modulates divergent downstream signaling pathways.

Cross-linking the BCR likely orchestrates multiple processes required for receptor internalization. The SFTKs can phosphorylate clathrin heavy chain enhancing its recruitment to the plasma membrane [ 66]. Other critical components of the endocytic machinery such as BAM-32 [ 16– 18], AP-2 [ 67], and Eps15 [ 68] are also regulated by phosphorylation. In addition, ligand-induced receptor conformational changes can facilitate receptor endocytosis by revealing cryptic AP-2 binding sites [ 69]. These, and other mechanisms [ 70], probably sequester ligated receptors into clathrin-coated vesicles.

Some receptors, such as the EGFR, continue to signal after internalization and this appears to be necessary for the efficient activation of selected MAP kinase pathways [ 35, 36]. Recent studies with pharmacological inhibitors of internalization have suggested that the BCR might function in a similar manner [ 18]. However, our observation that only non-phosphorylated BCR complexes are internalized argues that signaling occurs at the cell surface and does not persist after internalization. In support of this conclusion, inhibiting receptor internalization had no impact on distal MAP kinase pathway activation ( Figure 5). Furthermore, it is likely that the structural constraints imposed by intracellular trafficking preclude prolonged signaling from endocytic compartments. Internalized aggregated BCRs are rapidly delivered into multivesicular late endosomes [ 26]. In these compartments, the internalized BCR resides within intraluminal vesicles with the tails of Igα/Igβ separated from the cytosol by two limiting membranes.

Interestingly, phosphorylated receptor complexes were present in only a small minority of surface BCR patches. This corresponded to biochemical studies demonstrating that only a small fraction of surface BCR complexes were phosphorylated following ligation. Similar inefficiencies in the productive phosphorylation of TCRζ have been reported [ 71]. Phosphorylation of the BCR is initiated within 15 s of receptor ligation and reaches a maximum within 1–2 min [ 65, 72]. This is before clear patches are formed on the cell surface [ 73, 74]. Therefore, it is likely that unphosphorylated and phosphorylated receptors are sorted into different patches. This would be consistent with our model in which receptors, based on their phosphorylation status, would be functionally segregated into different groups. In regards to non-phosphorylated receptors, it is likely that surface aggregates are defined by localization to common clathrin cages [ 33, 37– 39]. The mechanisms resulting in the local concentrations of phosphorylated BCRs are not known. However, it is unlikely to simply arise from passive exclusion from clathrin-coated pits.

Retention of Igα/Igβ on the cell surface might be advantageous for propagating signals, but it would apparently impede the other function of the BCR, which is to capture antigen for processing and presentation to cognate T cells. However, it has been reported that following receptor stimulation, the BCR can become destabilized and that Igα/Igβ can dissociate from mIg [ 75]. Although originally reported in the context of anergy [ 76], it has recently been reported to occur in response to activating ligands [ 77]. Once disassociated, mIg and Igα/Igβ are endocytosed separately [ 77, 78]. Given the infrequency of inductive BCR phosphorylation, it is likely that only a small fraction of engaged BCRs are destabilized. This conclusion is consistent with recent elegant studies demonstrating that the bulk of surface BCR complexes remain intact following ligation [ 79]. However, for those receptors that have productively engaged antigen, dissociation into antigen binding and signaling subunits would allow each to adopt different subcellular fates better suited for their intrinsic function.

Mathematical analysis of the exclusive model allowed us to relate our findings to the wider body of experimental observations concerning the relationship between BCR signaling and internalization. This was particularly helpful in understanding how blocking endocytosis at different steps could result in dramatically different signaling phenotypes [ 33, 58] ( Figure 6B). Our analysis also provided insights into other observed behaviors of the BCR including the attenuation of signaling in response to high avidity ligands [ 80]. This aspect of BCR signaling may contribute to the observed in vivo ceilings in antibody affinity maturation [ 81].

Mathematical modeling also provided insights into the consequences of exclusive internalization and signaling that could not be obtained experimentally. This is because BCRs following either exclusive or non-exclusive mechanisms of internalization cannot be derived. However, such comparisons are necessary for understanding how a particular internalization mechanism could influence signaling in response to ligands of varying avidity ( Figure 6C).

Through the studies presented in this paper, we provide a new model for dissecting the complex dynamics of BCR internalization and signaling. As described above, this model may help explain seemingly disparate observations in the literature on how the BCR is spatially regulated. Furthermore, by identifying how receptors are selected for internalization, one can then begin to address how signaling is modulated by ligands of different avidity and how this translates into different cell fates during peripheral immune responses.

## Materials and Methods

### Plasmid construction and derivation of cell lines.

Construction of the platelet-derived growth factor receptor β (PDGFRβ)/Igα chimera has been previously described [ 23]. The tyrosine-to-alanine mutations of Igα were generated by QuickChange site-directed mutagenesis kit (Stratagene, La Jolla, California, United States). These chimeras and a dominant negative dynamin (K44A, S. Schmid, Scripps Research Institute, La Jolla, California, United States) were subcloned into the green fluorescent protein (GFP) containing retrovirus vector MIGR1 (H. Singh, University of Chicago, Chicago, Illinois, United States). Virus was produced in the packaging cell line GP293 (Clontech, Palo Alto, California, United States). 48 h later, supernants were collected and transfected into A20IIA1.6 cells. Cells were sorted for GFP expression by flow cytometry (MoFlo-HTS, DakoCytomation, Glostrup, Denmark). Surface expression of chimeric receptors was assayed by surface staining with mouse anti-hPDGFRβ antibodies (R&D Systems, Minneapolis, Minnesota, United States) followed by PE-conjugated anti-mouse IgG1 (BD Pharmingen, San Diego, California, United States). Samples were then examined by flow cytometry (FACScan, Becton Dickinson, Palo Alto, California, United States).

### Receptor internalization.

Cell aliquots (2 × 10 ^5^ cells/sample) were stimulated via the chimeric receptor by incubation with platelet derived growth factor-BB (PDGF-BB)(100 ng/ml; Sigma, St. Louis, Missouri, United States) for 5 min followed by anti-hPDGFRβ (5μg/ml) for 5 min. Cells were washed and then incubated with PE-conjugated anti-mouse IgG1 (5μg/ml) for 15 min on ice. Cells were then washed and resuspended in 100 μl of ice-cold FACS buffer (3% BSA in PBS), then incubated at 37 °C for the indicated intervals. To terminate internalization, cells were placed on ice. To assay BCR internalization, cells were stained with PE-conjugated anti-mouse IgG2a (5 μg/ml; BD Pharmingen) for 30 min on ice, then incubated at 37 °C for the indicated times. To remove surface-bound Ab, 300 μl of stripping buffer (100 mM glycine, 100 mM NaCl [pH 2.5]) was added to resuspend cells at room temperature for 2 min followed by washing in FACS buffer. In the indicated experiments, cells were pre-treated with Latrunculin (0.25 μg/ml, Molecular Probes, Eugene, Oregon, United States) at 37 °C for 1 h.

Fluorescence was measured using flow cytometry and receptor internalization was calculated using the following formula: % *SR*t = 100 × *(ARF*t- *AF)/(SF*- *AF)* where *SR*t is the percent of surface receptor internalized at time “t,” *ARF*t is the acid-resistant fluorescence at time “t,” *AF* indicates cellular autofluorescence of cells (median fluorescence of unstained cells or cells that were stained and then acid-stripped), and *SF* refers to the median fluorescence of cells that were stained for 30 min at 4 °C.

### Biochemical identification of internalized BCRs.

A20IIA1.6 cell aliquots were suspended at a concentration of 25 × 10 ^6^ cells/ml in ice-cold PBS. Cells were pulse-labeled by adding 80 μl of 10 mM Sulfo-NHS-SS-Biotin (Pierce, Rockford, Illinois, United States) per milliliter of reaction volume at 4 °C for 40 min. Cells were then washed with ice-cold phosphate buffered saline (PBS). Cells were incubated on ice with rabbit anti-mouse IgG (Jackson ImmunoResearch, West Grove, Pennsylvania, United States) at a final concentration of 5 μg/ml and then warmed to 37 °C for the indicated times. Surface retained biotin was removed using reduced glutathione (50 mM glutathione, 75 mM NaCl, 1 mM EDTA, 1% bovine serum albumin (BSA), 0.75% [vol/vol] 10 N NaOH). Cells were then washed with ice-cold PBS at 4 °C and lysed in 1 ml of NP-40 lysis buffer [ 47] for 30 min on ice. Lysates were clarified by centrifugation. Following pre-clearing with Protein A-Sepharose (Amersham, Little Chalfont, United Kingdom), supernatants were sequentially precipitated with streptavidin-Sepharose (Amersham) followed by anti-Igα antibodies [ 47] precoupled to protein A-Sepharose. Following washing, samples were boiled in Laemmli reducing SDS sample buffer, subjected to SDS-polyacrylamide gel electrophoresis (SDS-PAGE) and transferred to polyvinylidene difluoride (PVDF) membranes (Millipore, Billerica, Massachusetts, United States).

### Immunoprecipitations and immunoblotting.

Cell aliquots (1.5 × 10 ^6^ for whole cell lysates or 10 ^7^ for immunoprecipitations) were incubated with rabbit anti-mouse IgG (final concentration 20 μg/ml) for 5 min and then transferred to 37 °C for the indicated times. Stimulated cells were washed with ice-cold PBS and lysed in 1% NP-40 lysis buffer [ 47]. For immunoprecipitations, lysates were clarified by centrifugation, precleared with protein A-Sepharose, and then subjected to immunoprecipitation with protein A-Sepharose-coupled antibodies at 4 °C. Laemmli SDS reducing sample buffer was added to all samples, which were then subjected to SDS-PAGE followed by transfer to PVDF membranes, which were then blocked in 3% BSA-Tris-buffered saline with 1% Triton X-100 (TBST). Membranes were then incubated with the indicated antibodies, washed with TBST, incubated with horseradish peroxidase-labeled secondary antibodies (Amersham), and then washed with TBST. ECL (Amersham) was used to visualize immunoreactive proteins. To strip blots, membranes were incubated in TBST-0.4% SDS [pH 2.5] for 1 h at room temperature before being washed and reblotted as described above. Antibodies utilized included antiphosphotyrosine (4G10, Upstate Biotechnology, Lake Placid, New York, United States), anti-ERK (Zymed, Invitrogen), anti-phospho-ERK (Cell Signaling Technology, Massachusetts, United States), anti-JNK (BD Pharmingen), anti-phospho-JNK (Promega, Madison, Wisconsin, United States), anti-p38 (Santa Cruz Biotechnology, Santa Cruz, California, United States), anti-phospho-p38 (Cell Signaling), anti-PLCγ2 (Santa Cruz Biotechnology), and anti-Igβ extracellular domain (BD Pharmingen). The anti-Igα and anti-BLNK antibodies have been described previously [ 23].

### Confocal microscopy.

Spleens were isolated from BLNK ^−/−^ mice [ 53] and wild-type littermate controls. Following hypotonic lysis, cells were incubated with biotinylated anti-CD11b, anti-Grl, anti-CD3, anti-CD4, anti-CD8, anti-NK1.1, and anti-TER 119 (BD Pharmingen) on ice for 30 min, washed, then incubated with streptavidin microbeads (Miltenyi Biotec, Bergisch Gladbach, Germany) for 30 min at 4 °C. Following separation by MACS column (Miltenyi Biotec), purity of B cells was assayed by flow cytometry. Only populations of greater than 90% purity were used in experiments. To stimulate the BCR, cells were stained with FITC-conjugated goat anti-mouse IgG + IgM F(ab) _2_ (5 μg/ml, Jackson ImmunoResearch) on ice for 15 min. For BLNK knockdown A20IIA1.6 cells, BCR were incubated with Cy5 conjugated goat anti-mouse IgG (Jackson ImmunoResearch) on ice for 15 min. Cells were then warmed to 37 °C for the indicated times. Following fixation in 3% paraformaldehyde/3% sucrose/PBS, cells were permeabilized with 0.05% saponin and stained by PE-conjugated GST-BLNK-SH2 [ 23] on ice for 1 h. Images were collected using Leica (Wetzlar, Germany) SP2 AOBS LSM confocal microscope.

### Mathematical modeling.

In the model, the antigen, AP protein, and Syk proteins are treated implicitly, which corresponds to assuming that these species are in excess of their bound forms. The resulting equations for the exclusive model are:



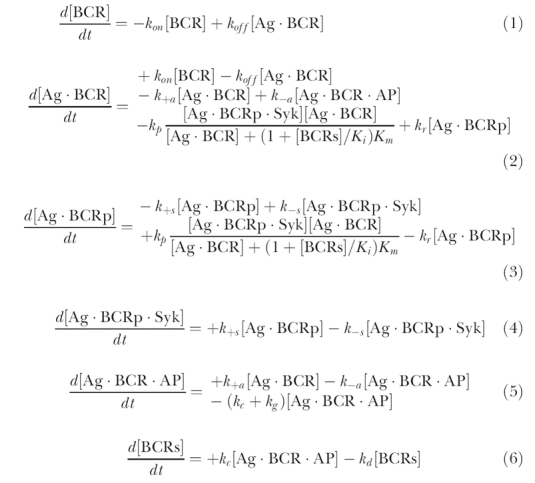



As described in Results, there are essentially two competing pathways: phosphorylation and internalization. The behavior of the model depends most sensitively on the parameters *k*_p_ and *k*_a_ because their relative sizes control how BCR partition between these reaction channels. We thus adjusted the values of *k*_p_ and *k*_a_ with remainder of the parameters set to values on the order of one, so that they were not rate-limiting; *k*_p_ was varied to make the peak in phosphorylation comparable to the observed level (about 10%), and *k*_a_ was varied to match the overall experimental time course of phosphorylation (about 10 min). The remaining parameters were then adjusted by trial-and-error to refine the wild-type phosphorylation profile for better agreement with the experimental data. Clearly, this procedure does not define a unique set of parameter values, but our objective in exploring the model is to obtain qualitative insight rather than to extract quantitative estimates for the kinetics of specific processes.

To validate the above procedure, we varied the rate constants from 0.1 to 100 min ^−1^ and examined the effects on the phosphorylation peak height and time ( Figure S2). In the case of reversible processes (binding, phosphorylation, and sequestration), the kinetic parameters for the forward and backward reactions were changed together to maintain their ratio; otherwise (removal), parameters were changed individually. The remaining parameters were held fixed at their values in the Figure 6 legend. Consistent with our expectations, we found that the peak was most sensitive (by a factor of two to three) to *k*_p_ and *k*_a_ (together with *k*_r_ and *k*_–a_).

Setting *k*_d_ = 0 corresponds to dn-dynamin and *k*_c_ = 0 to clathrin deletion. The trends for the mutants (decrease in phosphorylation for dn-dynamin and increase in phosphorylation for clathrin deletion) are robust to variations in the parameters given the ansatz that sequestered BCR inhibit further phosphorylation and a reasonable wild-type profile. This insensitivity to the choice of parameters derives from the fact that sequestered BCR appears only in the denominator of the phosphorylation rate law, so that raising [BCRs] always decreases *d*[Ag·BCRp]/ *dt,* and lowering [BCRs] always increases *d*[Ag·BCRp]/ *dt.* In turn, *d*[BCRs]/ *dt* depends monotonically on *k*_c_ and *k*_d_, but with opposite signs.

To obtain the non-exclusive model, it is necessary to add reaction channels corresponding to phosphorylation of the AP-associated receptor and AP binding to BCRp. This results in one additional reaction:



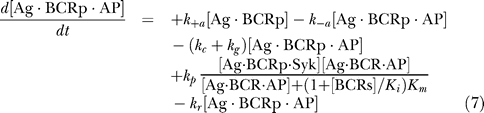



as well as two additional terms in Equation 5:



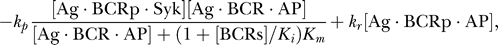



two additional terms in Equation 3:



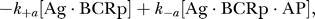



and one additional term in Equation 6:



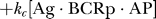



Simply adding the reaction channels indicated in the box in Figure 6A with the same values for the kinetic parameters as in the exclusive model results in very little phosphorylation at all times because phosphorylated BCRs are removed before they can activate Syk and propagate the signal. To meaningfully relate the exclusive and non-exclusive models, we thus adjusted one parameter in the latter such that the integrated phosphorylation was the same in both cases. The qualitative behavior we report was observed regardless of whether the phosphorylation rate was increased or the amount of sequestration was decreased ( Figure 6, legend). Because sequestered BCR still inhibit phosphorylation in the exclusive model, the mutant trends discussed above are reproduced by it as well. The equations were solved numerically with a fourth-order Runge-Kutta integrator [ 82].

## Supporting Information

Figure S1 Knockdown of BLNK in A20IIA1.6 Cells (A) Total cell lysates from cells expressing a BLNK specific shRNA, or vector alone, were immunoblotted with anti-BLNK and anti-Igα antibodies. (B) Indicated cells were stimulated with anti-IgG for 3 min and total cell lysates immunblotted with anti-phosphotyrosine antibodies (4G10). Position of BLNK indicated (*). (C) Cells expressing indicated shRNA were stimulated with anti-IgG (3 min), lysed, and PLCγ2 immunoprecipitated. Samples were resolved by SDS-PAGE and probed sequentially with 4G10 and anti-PLCγ2 antibodies.
Methods: BLNK knockdown cell line was made by shRNAs targeting nucleotides 909–937 and 1054–1081 in the BLNK open reading frame. The Hannon algorithm was used to select and design shRNAs to target the BLNK open reading frame ( http://katahdin.cshl.org:9331/RNAi). PCR was performed on a pGEM vector containing the U6/SP6 promoter with primer sets encoding shRNAs predicted to target BLNK. The resulting 500 base pair fragments were cloned into pENTR/D-TOPO vector (Invitrogen, Carlsbad, California, United States) and then shuttled into MSCV PIG (puromycin/IRES/GFP) Gateway vector (Invitrogen). A20IIA1.6 cells were transfected with these constructs by using retrovirus collected from packaging cell line GP-293 (Clontech), and high GFP clones were selected by flow cytometry (MoFlo).
(1.2 MB TIF)

Figure S2 Sensitivity of Model Results to Parameter Values Variation in the (A) height and (B) time of the peak in phosphorylation as rate constants indicated were varied from 0.1 to 100 min ^−1^ with the remainder fixed at their values (given in the legend to Figure 6 in the main text). The ratios *k*_p_/ *k*_r_, *k*_+a_/ *k*_–a_, *k*_+s_/ *k*_–s_, and *k*_on_/ *k*_off_ were held fixed.
(747 KB EPS)

## Abbreviations

AP: adaptor protein
BCR: B cell antigen receptor
ITAM: immunoreceptor tyrosine-based activation motif
MAP: mitogen-activated protein
PDGFR: platelet derived growth factor receptor
PE: phycoerythrin
SFTK: Src-family tyrosine kinase
